# Significant value of XRCC2 and XRCC9 expression in the prognosis of human ovarian carcinoma

**DOI:** 10.7150/jca.59273

**Published:** 2021-08-28

**Authors:** Yi Liu, Yichi Xu, Mengying Jiang, Weinan Chen, Xueqiong Zhu

**Affiliations:** 1 Department of obstetrics and gynecology, the Second Affiliated Hospital of Wenzhou Medical University, Wenzhou, China; 2 Department of Pathology, Beth Israel Deaconess Medical Center, Harvard Medical School, Boston, MA, 02215, USA

**Keywords:** XRCC2, XRCC4, XRCC9, Ovarian carcinomas, Prognosis

## Abstract

**Background:** The x-ray repair cross-complementing (XRCC) family is essential in DNA repair processes. The predictive roles of XRCCs remain unclear in ovarian carcinomas. Therefore, detecting the relationship between XRCCs expression and ovarian carcinomas prognosis is increasingly pivotal.

**Methods:** Using the “Kaplan-Meier (KM) plotter” database, progression-free survival (PFS) and overall survival (OS) were utilized to evaluate the prognosis of XRCCs mRNA expression in ovarian carcinoma patients with clinical outcomes. Then, mRNA level and protein levels of XRCCs were assessed in normal ovarian cells and ovarian carcinoma cell lines by real-time qPCR, Western blotting and immunofluorescence analysis. Additionally, expression of the XRCCs protein in tissues from ovarian carcinomas and normal ovary was identified by immunohistochemical staining.

**Results:** Higher mRNA levels of XRCC2 and XRCC9 predicted longer PFS and OS in all women with ovarian malignance, while elevated XRCC4 mRNA levels were linked to poor PFS and OS in all ovarian cancer patients. Elevated mRNA of XRCC2 was also correlated with better PFS in patients with serous ovarian carcinomas, and better PFS and OS in grade III and stage III+IV ovarian carcinomas patients. What's more, highly expressed levels of XRCC9 mRNA were also linked to favorable PFS and OS in patients with serous, grade III and stage III+IV ovarian carcinomas. Nevertheless, elevated mRNA expression of XRCC4 was linked to worse PFS and OS for patients with serous, grade III as well as all stages of ovarian malignance. Additionally, when compared to ovarian carcinoma cell lines, elevated mRNA and protein levels of XRCC2 and XRCC9 were detected in normal ovarian cells. Consistently, higher staining of XRCC2 and XRCC9 was also detected in normal ovarian cells than that in ovarian cancer cells. Then, higher staining levels of XRCC2 and XRCC9 were discovered in healthy control tissues than that in ovarian carcinoma tissues. Meanwhile, XRCC4 was identified to be overexpressed in tissues of ovarian malignance as compared to normal control tissues. However, XRCC4 mRNA and protein levels were lower in ovarian cancer cells than that in normal cell line.

**Conclusion:** Elevated XRCC2 and XRCC9 expression levels were observed in normal ovarian cells and tissues than that in ovarian malignance cells and tissues, and exhibited better prognostic value especially in patients with serous, poor differentiated and late stage, suggesting that XRCC2 and XRCC9 may be potent prognostic markers in ovarian cancer patients and can guide personalized surveillance for ovarian malignance.

## Background

Ovarian carcinoma is one of the common causes of cancer death in women in the world 1. In the United States, more than twenty thousand patients with ovarian carcinoma will be diagnosed and over ten thousand cases will die due to this disease in 2021 as estimated 2. Many studies have demonstrated that surgical cytoreduction accompanied with subsequent platinum-based chemotherapy is optimal treatment for advanced ovarian cancer 3. Although great efforts have been made to make progressions in early detection and treatment, population-based incidence and mortality of ovarian cancer remain high 4. Further research focusing on the update of new biomarkers to predict the prognosis, in some ways, will be likely to enhance the survival of ovarian carcinoma patients for progression-free survival (PFS) and overall survival (OS) in ovarian carcinoma patients.

The XRCC family is composed of ten sub-classes (XRCC1-9 and XRCC11) 4. XRCC genes have been identified as pivotal factors in the protection of mammalian cells from ionizing radiation damages 5. Furthermore, XRCC genes have also implicated in the processes of DNA repair in mammalian, particularly in double-strand break repair, which perhaps participate into gynecological tumors 6-8.

In addition to the importance of XRCCs in DNA repair, XRCCs have been identified as significant factors in the prognostic value of various solid tumors 9-11. Nevertheless, at the transcriptional expression levels, the prognosis of individual XRCC gene in patients with ovarian carcinoma remains unknown. In the present research, we explored the prognostic value of XRCC family with various clinical features. Furthermore, the transcriptional level and protein expression of some XRCCs were also assessed in normal ovarian cells and tissues and ovarian cancer cells and tissues. Therefore, our aim is to detect the significant prognostic role of some XRCCs, which may guide personalized ovarian cancer surveillance.

## Methods

### The Kaplan-Meier Plotter

KM plotter, an online database 12, was utilized to detect the OS as well as PFS of XRCC genes at transcriptional level (http://kmplot.com/analysis/). Notably, this database was capable of assessing the prognostic value of multiple genes in ovarian carcinoma patients with average follow-up time of 40 months. The background of databases contained breast carcinomas 12, lung carcinomas, and gastric as well as ovarian carcinomas 13. In addition, clinical data of patients, including TP53 mutation status, grade, stage, histology and applied chemotherapy, was also involved in KM plotter. Shortly, the XRCC family was searched in the KM plotter database to obtain the PFS and OS. The samples were separated into 'low' and 'high' groups via established cutoffs. The Kaplan-Meier survival plot, 95% confidence intervals (CIs), hazard ratio (HR), and log-rank *P* value were displayed on the webpage.

### Cell lines and cell culture

The immortalized ovarian surface epithelial cell line IOSE, ovarian carcinomas cell lines ES2 and OVCAR3 were obtained from European Collection of Authenticated Cell Cultures and cultured in Dulbecco's modified Eagle's medium (DMEM) (Gibco, USA) with 10% fetal bovine serum (Gibco, Thermo Fisher Scientific) and 1% penicillin and streptomycin (Gibco, Thermo Fisher Scientific). Then the cells were incubated at 37˚C with 5% CO_2_.

### Real-time qPCR (RT-qPCR)

Cell RNA was obtained via TRIzol reagent (Invitrogen, Thermo Fisher Scientific). Using Real-time PCR SYBR Master mix (Applied Biosystems), the cDNA was synthesized and qPCR was conducted. Then, gene expressions were performed by LightCycler 480 II real-time PCR (Roche). The reaction mixture contained 1X SYBR RT‐PCR buffer, MgCl_2_, cDNA template and primer, and conditions were 95˚C for 55 sec, 10 min hold for 40 cycles at 95°C, 30 sec extensions at 60˚C, then incubated for 1 min at 60°C, which was normalized with GAPDH using the 2^-ΔΔCt^ method. The primes of XRCC2, F: TAAGGCATGGCAGCAAC, R: CAACCCCACTTTCTCCAA. XRCC4, F: TTGGGAGAAAACACTGGAA, R: TCATCAGCTTCTTGGGAAA. XRCC9, F: CTGCCTGGACCTGTGGA, R: GGGAGCCCTTGCAGACTA. GAPDH, F: AGGTCGGTGTGAACGGATTTG, R: GGGGTCGTTGATGGCAACA.

### Western Blotting

Total protein was extract by Radioimmunoprecipitation assay buffer (Beyotime Biotechnology, Shanghai, China) after washing cell lines in 10 cm dish three times with PBS. Then samples were heated at 100°C for 10 min and loaded into SDS-polyacrylamide gel (10%). Followed with electro-blotted protein onto polyvinylidene (PVDF) membranes, and then using 5% non-fat dry milk to block. Then using TBST washed the membranes and incubated with anti-XRCC2 (Abcam, Rabbit anti-human polyclonal, ab180752, 1:500), anti-XRCC4 (Proteintech, Rabbit anti-human polyclonal, 15817-1-AP, 1:1000), anti-XRCC9 (Abcam, Goat anti-human polyclonal Abcam, ab115230, 1:1000), and anti-GAPDH (Abcam, Rabbit anti-humanmonoclonal, ab181602, 1:2000,) at 4˚C overnight. Then after washed and incubated with the second anti-rabbit or anti-goat antibody for 1h, the membranes were used enhanced chemiluminescence reagent (Millipore, USA) and detected though a software (Bio-Rad, Hercules, USA). Each experiment was repeated three times.

### Immunofluorescence staining (IF)

The IOSE, ES2 and OVCAR3 cells were seeded at 3×10^5^/well in a 6-well cell plate with small glass slides and cultured overnight. Cells were washed with PBS and fixed with paraformaldehyde (4%) for 30 min at room temperature. Then, washing cells three times with PBS and 0.1% Triton/PBS for 10 min. After blocking cells with blocking buffer (2% BSA/0.1%Tween20/PBS) for 30 min at room temperature. Then incubating cells with rabbit XRCC2 (Abcam, ab180752, 1:100 dilution) and rabbit XRCC4 (Abclonal, A18046, 1:50 dilution) and rabbit XRCC9 (Proteintech,10215-1-AP, 1:50 dilution) overnight at 4°C. After being washed with PBS, the smears of cells were stained with FITC-conjugated anti-rabbit IgG antibodies at room temperature for 1 h. Then stained with DAPI, aqueous, fluoroshield mounting medium (Abcam, ab104139). The slides were observed and imaged using a fluorescence microscope, and calculated by ImageJ.

### Patients and tissue samples

All 15 ovarian carcinoma tissue slices and 15 healthy ovarian slices were obtained from the Second Affiliated Hospital of Wenzhou Medical University between 2017 and 2019. Furthermore, ovarian malignant paraffin embedded slides were taken from ovarian carcinoma patients pathologically diagnosed after surgery. And normal control tissues came from patients with unilateral ovarian benign lesions who underwent bilateral oophorectomy. The patients have signed an informed consent form before the operation. The ethics committee of the Second Affiliated Hospital of Wenzhou Medical University supports this study.

### Immunohistochemical analysis (IHC)

Human ovarian malignant tissues and normal healthy ovary tissues were heated 1 h at 65˚C for deparaffinization and then incubated in sodium citrate buffer with microwave 20 min. Then washing the sections twice with PBS and bovine serum albumin (3%) blocked for 40 min and incubated with primary antibodies (XRCC2, ab180752, 1:100; XRCC4, 15817-1-AP, 1:100; XRCC9, ab115230, 1:100) overnight at 4°C. Next day, goat anti-rabbit or donkey anti-goat antibodies (ZSGB-BIO, China) were used as second antibodies. Then, followed 3,3'-Diaminobenzidine staining and hematoxylin counter-staining, the sections were dehydrated via xylene, mounted and cover slipped. Negative control and positive control were set in every test. The staining level was based on semi-quantitative immunohistochemical detection, which multiplied the staining intensity score (negative for 0, weak for 1, moderate for 2, strong for 3) and the percentage score (positive cells 1-25% for 1, 26-50% for 2, 51-75% for 3, >75% for 4). The analysis of IHC images was in a blind manner. The pathological results of these IHC images from the normal ovarian tissues and ovarian cancer tissues were unclear for Y.L. and Y.X. And the evaluation and protein staining quantification of IHC images were performed independently by Y.L. and Y.X. Then samples were individually discussed until consensus was reached.

### Statistical analysis

Data analysis was processed with Graph Pad Prism 6.0 software and assessed via using unpaired t-tests with Welch's correction. Data were displayed as mean ± standard deviation. It was recognized as statistically significance when *P* value <0.05.

## Results

In order to investigate expression of XRCCs at mRNA levels in ovarian cancer patients, analyses focusing on the prognostic function of individual XRCC family via the KM plotter were conducted. All XRCC family members could be found in the database except XRCC8 and XRCC11, probably due to the low level of XRCC8 and XRCC11 in tissues. Considering the fact that molecular events, risk factors, therapeutic targets, and prognostic markers might vary in different subtypes of ovarian carcinomas, the significant prognostic value of XRCC family was then evaluated in all ovarian carcinoma patients.

### The PFS and OS of XRCCs in all ovarian carcinoma patients

**Figure 1:**
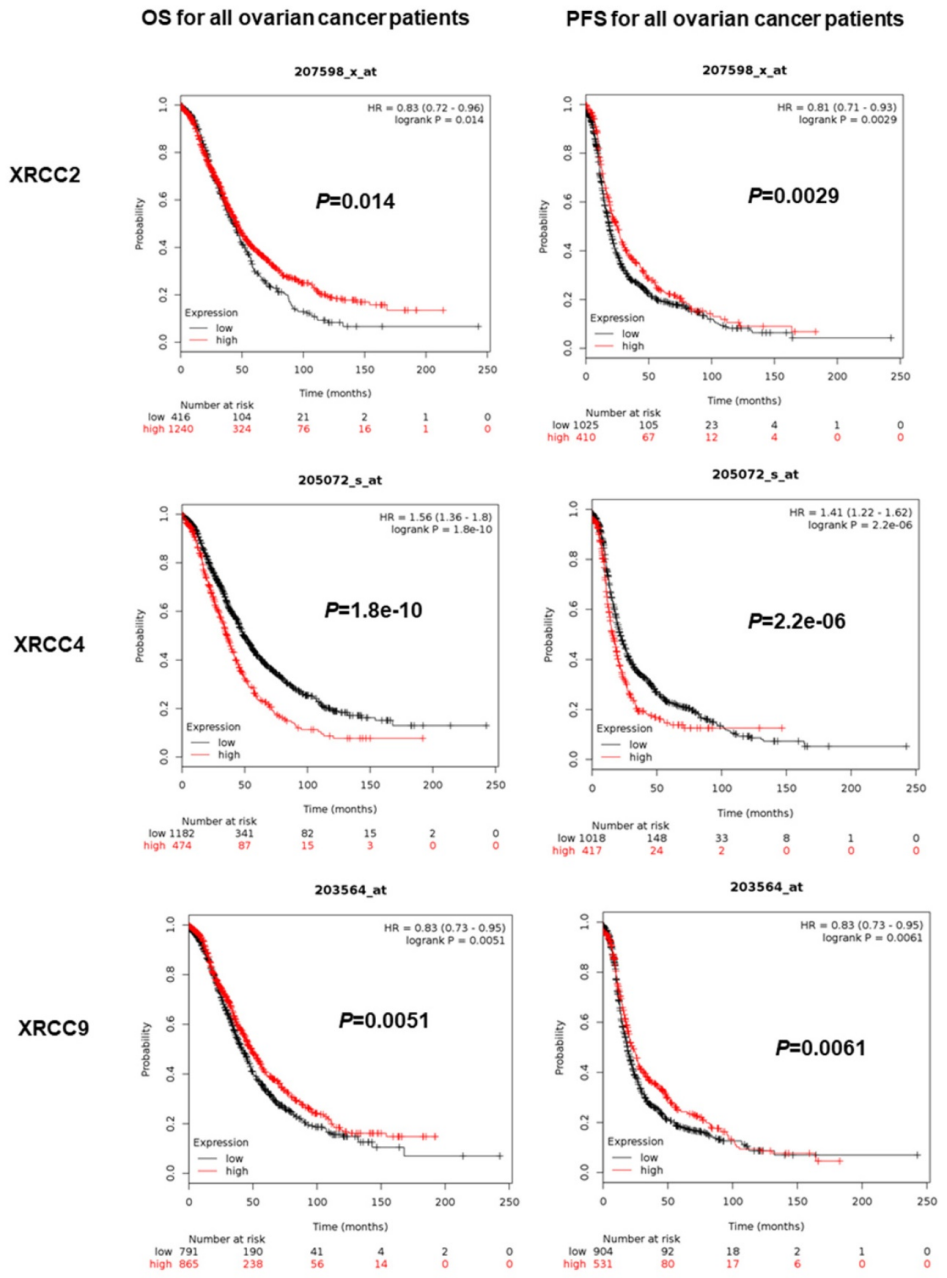
** The prognostic significance of XRCCs in all ovarian carcinoma patients.** The OS and PFS of XRCC2, XRCC4 and XRCC9 for all ovarian carcinoma patients.

The prognostic value of eight XRCCs was firstly determined with PFS and OS for all ovarian carcinoma women. As shown in Figure 1, up-regulation of XRCC2 and XRCC9 was dramatically correlated with favorable PFS and OS for all ovarian carcinoma patients (XRCC2, PFS: HR=0.81 (0.71-0.93), *P*=0.0029; OS: HR=0.83 (0.72-0.96), *P*=0.014; XRCC9, PFS: HR=0.83 (0.73-0.95), *P*=0.0061; OS: HR=0.83 (0.73-0.95), *P*=0.0051). Furthermore, higher expression of XRCC4 was significantly linked to worse PFS and OS for all ovarian carcinoma women (PFS: HR=1.41 (1.22-1.62), OS: HR=1.56 (1.36-1.8), *P*=0.0000, *P*=0.0000). Interestingly, highly expressed XRCC6 was only linked to prolonged OS for all ovarian carcinoma patients, HR=0.81 (0.7-0.93), *P*=0.0022, but showed a null association with PFS. Additionally, high mRNA levels of XRCC1 and XRCC7 were only linked to a poor PFS in ovarian carcinoma patients (XRCC1: HR=1.29 (1.12-1.48), *P*=0.0004; XRCC7: HR=1.3 (1.14-1.48),* P*=0.0000), and were not linked to OS in all ovarian malignance patients. Nevertheless, the mRNA levels of XRCC3 and XRCC5 were uncorrelated to PFS and OS in ovarian carcinoma patients.

With these in mind, only XRCC2, XRCC4 and XRCC9 exhibited consistent results in PFS and OS for all ovarian carcinoma patients. Therefore, XRCC2, XRCC4 and XRCC9 were conducted for further research in various subtypes.

### Prognostic significance of XRCC2, XRCC4 and XRCC9 in serous and endometrioid ovarian carcinomas patients

**Figure 2:**
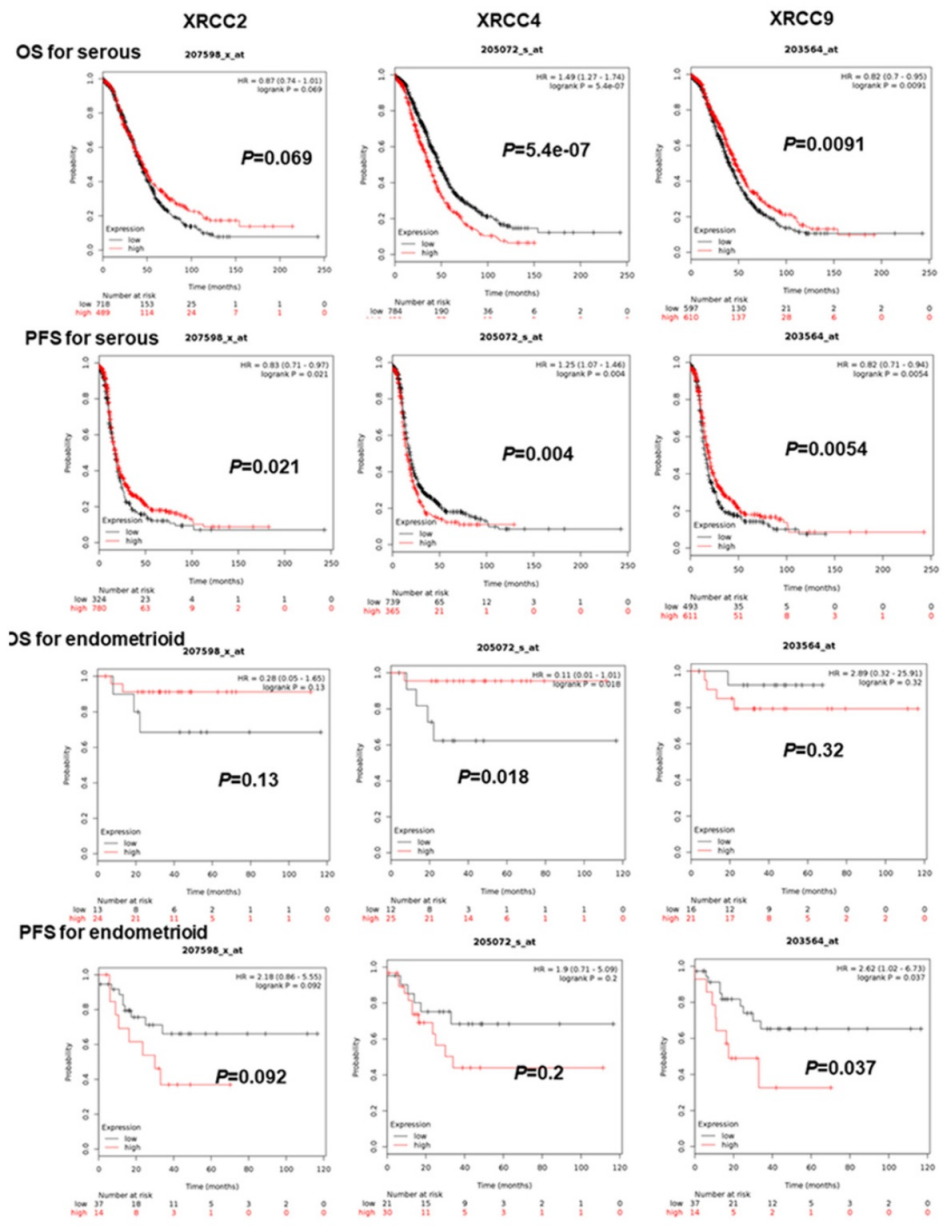
** The prognostic significance of XRCCs in serous and endometrioid ovarian carcinoma patients.** The OS and PFS of XRCC2, XRCC4 and XRCC9 for serous ovarian carcinoma patients and for endometrioid ovarian carcinoma patients, respectively.

As for further evaluating the association of XRCC2, XRCC4 and XRCC9 with other clinical features, the PFS and OS of these three genes were assessed in patients with serous and endometrioid ovarian malignance. As shown in Figure 2, increased XRCC2 mRNA was linked to prolonged PFS for serous ovarian carcinoma women, HR=0.83 (0.71-0.97), *P*=0.021, but was not linked to OS for serous or endometrioid ovarian carcinoma patients. Higher mRNA level of XRCC2 was also not linked to PFS for endometrioid ovarian carcinoma patients. Then, for XRCC4, overexpression of these genes was dramatically linked to worse PFS and OS for patients with serous ovarian carcinoma, HR=1.25 (1.07-1.46), *P*=0.004, HR=1.49 (1.27-1.74), *P*=0.0000. While for endometrioid ovarian cancer patients, it was linked to an improved OS, HR=0.11 (0.01-1.01), *P*=0.0182, but was not associated with PFS. Notably, elevated XRCC9 mRNA level was significantly linked to favorable OS as well as PFS in serous ovarian cancer women, HR=0.82 (0.7-0.95), *P*=0.0091, HR=0.82 (0.71-0.94), *P*=0.0054. But in endometrioid ovarian carcinomas patients, higher mRNA level of XRCC9 was correlated to poor PFS, HR=2.62 (1.02-6.73), *P*=0.037, and was uncorrelated to OS.

### Prognostic value of XRCC2, XRCC4 and XRCC9 in ovarian carcinoma patients with different pathological grades

Then, description of survival withXRCC2, XRCC4 and XRCC9 was conducted in ovarian malignant patients with different clinical grades. As shown in Table 1, increased XRCC2 showed a prolonged PFS in ovarian carcinoma patients with grade I (HR=0.17 (0.05-0.56), *P*=0.0009), as well as a better PFS and OS in grade III ovarian carcinoma patients (PFS: HR=0.76 (0.63-0.91), 0.0026; OS: HR=0.8 (0.67-0.96), *P*=0.016). For XRCC4, its higher level was involved in a worse OS in grade I (HR=3.92 (1.42-10.85), *P*=0.0049), also a worse PFS and OS in grade III ovarian carcinoma women (PFS: HR=1.38 (1.16-1.65), *P*=0.0004; OS: HR=1.56 (1.32-1.85), *P*=0.0000). What's more, the survival of XRCC9 was only associated with grade III patients, which showed a favorable PFS and OS in grade III ovarian carcinoma patients (PFS: HR=0.73 (0.62-0.86), *P*=0.0002, OS: HR=0.74 (0.62-0.87), *P*=0.0003).

### Prognostic value of XRCC2, XRCC4 and XRCC9 in ovarian carcinoma patients with various clinical stages

With regard to stages of ovarian carcinoma (Table 2), both XRCC2 and XRCC9 with high expression were considered as significantly favorable productive factors of PFS and OS in stage III+IV ovarian carcinoma patients (XRCC2, PFS: HR=0.78 (0.67-0.9), *P*=0.0010, OS: HR=0.72 (0.6-0.85), *P*=0.0000; XRCC9, PFS: HR=0.77 (0.67-0.88), *P*=0.0003, OS: HR=0.79 (0.68-0.92), *P*=0.0022). However, high level of XRCC2 was found to be involved in a poor PFS in stage I+II ovarian carcinoma patients (HR=2.11 (1.19-3.76), *P*=0.0093). Remarkably, highly expressed mRNA level of XRCC4 was linked to worse PFS and OS in ovarian carcinoma women with all stages (stage I+II: PFS: HR=1.77 (1-3.14), *P*=0.046, OS: HR=2.6 (1.2-5.65), *P*=0.012; stage III+IV, PFS: HR=1.23 (1.06-1.43), *P*=0.0072, OS: HR=1.53 (1.31-1.79), *P*=0.0000).

### The mRNA and protein expression of XRCC2, XRCC4 and XRCC9 in normal ovarian cells and ovarian carcinoma cells

**Figure 3:**
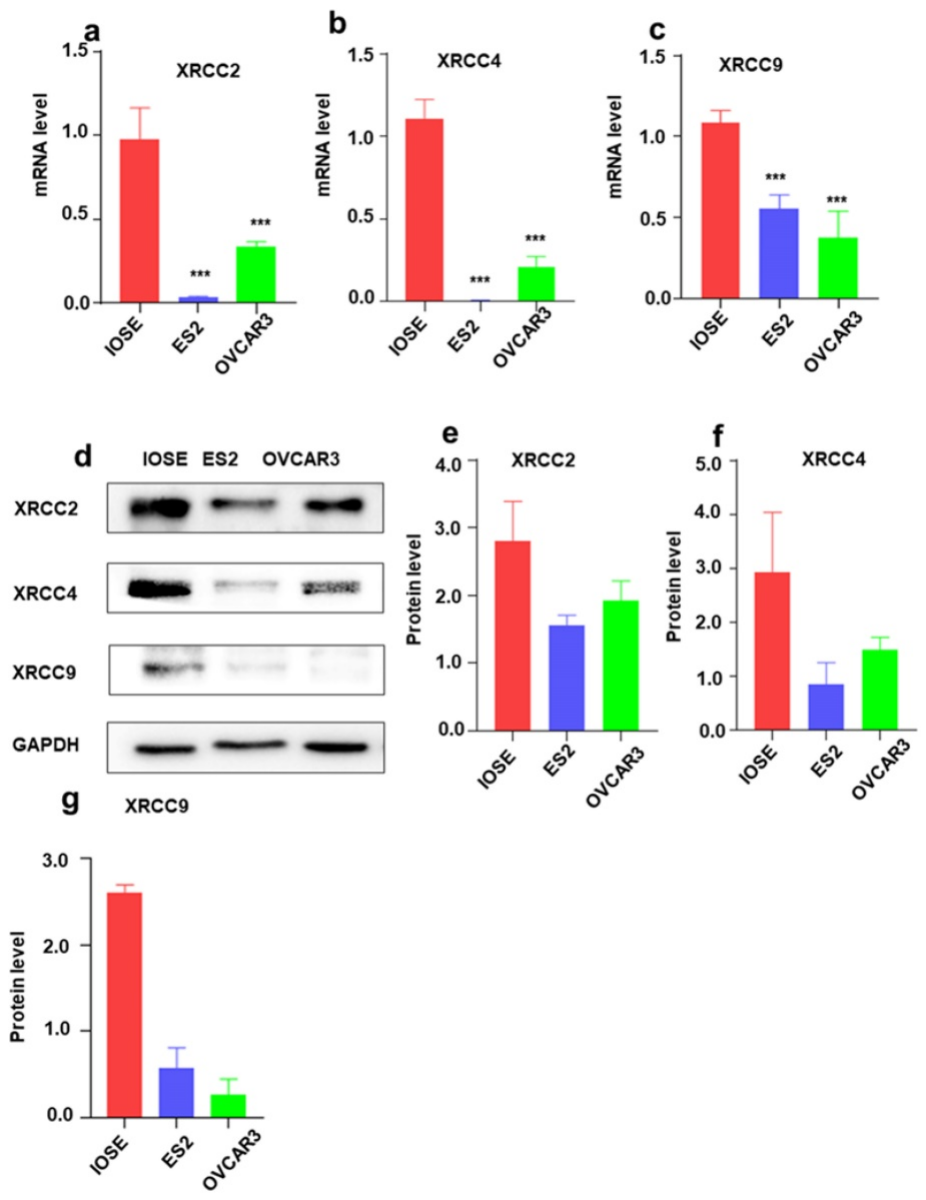
** The mRNA and protein expressions of XRCC2, XRCC4 and XRCC9 in ovarian carcinoma cells and normal ovarian cells.** The IOSE is normal ovarian cell lines. ES2 and OVCAR3 are ovarian malignant cell lines. a-c: The mRNA level of XRCC2, XRCC4 and XRCC9 in different cells. d-g: The protein expression of XRCC2, XRCC4 and XRCC9 in different cells were processed by Western blotting. *** *P* <0.001, data were triplicate and displayed as mean ± SD.

**Figure 4:**
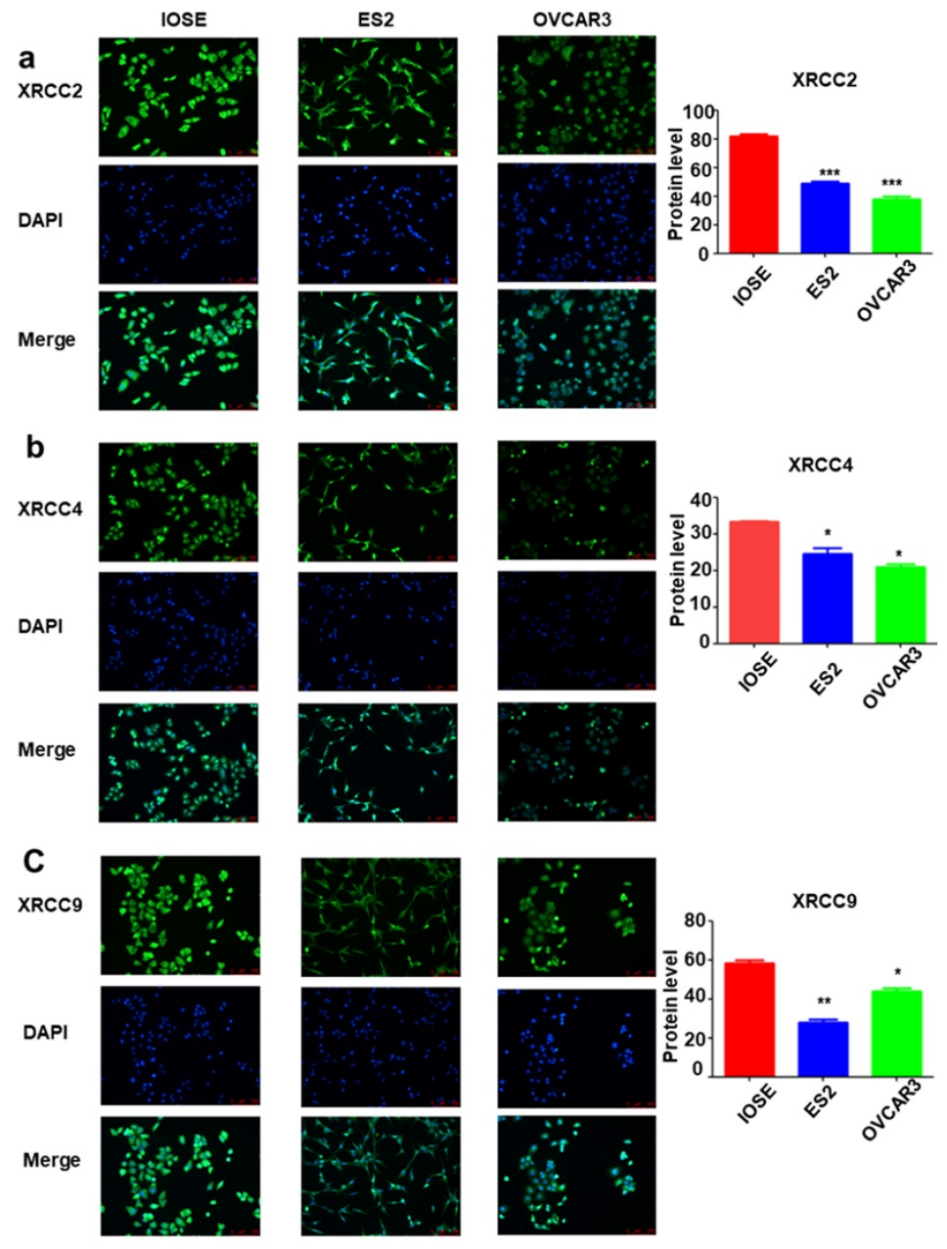
** The distribution of XRCC2, XRCC4 and XRCC9 in ovarian carcinoma cells and normal ovarian cells.** a-c: The distribution of XRCC2, XRCC4 and XRCC9 in IOSE, ES2 and OVCAR3 cells were processed by immunofluorescence. Green is the protein distribution, and blue is the nucleus. *** *P* <0.001, ** *P* <0.01, * *P* <0.05, data were triplicate and displayed as mean ± SD.

To further identify the expression of XRCC2, XRCC4 and XRCC9 genes in ovarian carcinoma cell lines, the mRNA level and protein level of these three genes were assessed. As shown in Figure 3, the results displayed that XRCC2, XRCC4 and XRCC9 mRNA levels were highly expressed in normal ovarian IOSE cells than in ovarian carcinoma ES2 and OVCAR3 cells (Figure 3a-c). Additionally, the protein levels of XRCC2, XRCC4 and XRCC9 were also showed higher expression in IOSE cells as compared with ES2 and OVCAR3 ovarian cancer cells (Figure 3d-g), which was corresponding to the results of mRNA. Consistently, immunofluorescence analysis observed higher staining of XRCC2, XRCC4 and XRCC9 in normal ovarian IOSE cells than that in ovarian carcinomas ES2 cells and OVCAR3 cells (Figure 4a-c). Furthermore, XRCC2 was located at nucleoplasm and vesicles (Figure 4a), XRCC4 was located at nucleus and XRCC9 was located at cytoplasm and nucleus.

### The protein level of XRCC2, XRCC4 and XRCC9 in tissues from ovarian carcinomas and normal ovary

To further validate the levels of XRCC2, XRCC4 and XRCC9 in tissues from normal ovary and ovarian carcinoma patients, IHC analyses were conducted to identify the protein expression differences among them. Significantly elevated protein levels of XRCC2 were observed in healthy control tissues, while they were rarely expressed in ovarian cancer tissues, the mean IHC score was 7.93±2.87 in normal ovarian tissues and 2.40±2.13 in ovarian cancer tissues, *P*<0.0001 (Figure 4a). Furthermore, XRCC4 was overexpressed in ovarian carcinomas samples and lowly expressed in normal ovarian samples, and the mean IHC score was 6.00±2.07 in ovarian cancer tissues and 2.13±1.51 in healthy tissues, *P*<0.0001 (Figure 4b). Then, the protein level of XRCC9 was detected to be highly expressed in tissues from normal ovary than tissues from ovarian carcinoma patients, and the mean IHC score was 7.40±2.64 in normal ovarian tissues and 3.13±1.77 in ovarian cancer tissues, *P*<0.0001 (Figure 4c).

## Discussion

The significant role of DNA repair gene polymorphisms in tumorigenesis has drawn an increasing attention nowadays. In our present study, via using the KM plotter database, the predictive value of XRCCs was systematically detected in patients with ovarian carcinomas. Our results discovered that elevation of XRCC2 and XRCC9 expression was linked to the favorable PFS and OS in all ovarian carcinoma patients. Moreover, elevated XRCC2 mRNA level was also linked to better PFS for patients with serous ovarian carcinomas, better PFS and OS for patients with grade III and stage III+IV ovarian carcinomas. Notably, higher level of XRCC9 was also involved in favorable PFS and OS in ovarian carcinoma patients with serous, grade III and stage III+IV. Conversely, highly expressed level of XRCC4 was linked to poor PFS and OS for all ovarian carcinoma patients, and predicted worse PFS and OS for serous, grade III as well as all stages ovarian carcinoma patients. Furthermore, XRCC2, XRCC4 and XRCC9 displayed elevated mRNA and protein levels in normal ovarian cell line, as compared to ovarian carcinoma cell lines. Notably, the protein level of XRCC2 and XRCC9 were found to be overexpressed in normal ovarian tissues, whereas XRCC4 was highly expressed in patients with ovarian carcinoma, which was consistent with the prognosis of ovarian cancer.

XRCC2, a crucial factor for homologous recombination 14, is also a DNA repair gene and could be connected to various cancers 15, 16. An elegant study has identified XRCC2 as a potential tumor-suppressor gene in mammals 17. However, XRCC2 might have diverse roles in progression and tumorigenesis of ovarian carcinoma, and the results of previous researches were inconsistent. Yuan and colleagues 18 failed to find a significant involvement between ovarian cancer risk and XRCC2 rs3218536 polymorphism through a meta-analysis recruiting 5802 cases and 9390 controls. But most recently, one study has reported that XRCC2 rs3218536 was significantly linked to risk of ovarian carcinoma under dominant contrast (AA+AG vs. GG) in overall population, especially among Caucasians 19. Similarly, Michalska and colleagues 20 conducted an experiment with 700 ovarian carcinoma patients and 700 healthy subjects, and revealed a significant increase of the XRCC2 188Arg/His and 188His/His heterozygote frequencies in ovarian carcinomas than that in healthy subjects, which indicated that XRCC2 Arg188His polymorphisms might be positively linked to ovarian malignances in Polish population, especially in grade I ovarian carcinomas. However, a meta-analysis by He et al. 21 demonstrated a completely opposite conclusion that XRCC2 rs3218536 polymorphism might be most closely linked to decreased risk of developing ovarian carcinomas. In this study, higher expression of XRCC2 mRNA and protein levels was observed in normal cells than that in ovarian carcinoma cell lines. Additionally, XRCC2 was also lowly expressed in ovarian cancer tissues, and indicated a better survival for ovarian malignance patients, However, in stage I and II ovarian carcinoma, the high XRCC2 expression was linked with a poorer PFS, which may be due to the different role of XRCC2 gene in early stage and late stage. To date, our findings display the expression level and predictive value of XRCC2 in ovarian carcinoma, implying that XRCC2 might be a favorable prognostic indicator for ovarian cancer, particularly in patients with serous, poor-differentiated and late stage.

XRCC4 participates in the process of Ku70/Ku80 heterodimer coupled with DNA ligase IV to act as an essential marker in DNA double-strand break repair 7, 8. XRCC4 was well studied in diverse human tumors such as lung carcinoma 22 and bladder carcinoma 23, which was reported as a poor productive factor in these cancers. p53 has been identified as an essential role in the XRCC4 associated double-strand break repair in the neural development 24. Additionally, the mutation of p53 phosphorylation at Ser18 and 23 (p53S18/23A) could completely rescue embryonic lethality of XRCC4-deficient mice and was able to suppress tumorigenesis in XRCC4-deficient mice 25. And ablation of XRCC4 in combination with p53 will induce brain tumors efficiently 26. These results imply that XRCC4 depletion is more likely to induce p53-dependent cell death in ovarian cancer. Interestingly, the function of XRCC4 in ovarian carcinoma remains mysterious. One paper by Willis et al. 27 recommended the increased expression of XRCC4 might be a prognostic factor predicted poor outcome in ovarian serous cancer. In line with this meta-analysis, our results discovered higher expression of XRCC4 protein in tissues from ovarian carcinoma patients than that from healthy controls, and elevated level of XRCC4 mRNA was significantly linked to worse survival for ovarian malignance patients, particularly in serous, grade III and all stages, implying XRCC4 might be a poor prognostic biomarker in ovarian carcinomas. Nevertheless, the high XRCC4 expression was correlated with a favorable OS in endometrioid ovarian carcinomas, which may be attributed to the less number of cases of endometrioid ovarian carcinomas in the database. Another reason may be that XRCC4 is more likely to play a different role in different pathological types of ovarian cancer. Therefore, XRCC4 may not be used as a clear indicator of the prognosis of ovarian cancer. However, the transcriptional level and protein level in normal ovarian cells were elevated than ovarian carcinomas cells, which were not consistent with the tissue results. The reason for this phenomenon may be attributed to the immortalization of cells affecting gene expression.

Compared with XRCCs mentioned above, the function of XRCC9 in malignancies is little known. It has been observed that XRCC9 (Thr297Ile) gene was correlated to a low risk of development of lung carcinomas, implying that XRCC9 may act as a protective gene in non-small cell lung carcinoma 28. In the present study, higher mRNA and protein levels of XRCC9 were observed in normal ovarian cell and tissues as compared to ovarian carcinoma cell lines and tissues, suggesting XRCC9 plays a pivotal role in the progression of ovarian cancer. In addition, our research discovered that higher mRNA level of XRCC9 predicted the better survival for all ovarian carcinoma patients, particularly in patients with serous, poor-differentiated and late-stage. Therefore, XRCC9 is more likely to be a protective prognostic biomarker for ovarian carcinoma.

## Conclusion

In summary, this study illustrated that elevated mRNA and protein levels of XRCC2 and XRCC9 were obviously detected in normal ovarian cells or tissues than that in ovarian carcinoma cells or tissues. Furthermore, XRCC2 and XRCC9 showed favorable predictive values in ovarian carcinoma, especially in patients with serous, poor-differentiated and late-stage. These results indicated that XRCC2 and XRCC9 might be potential biomarkers in ovarian carcinoma and help to predict the prognosis of patients with ovarian malignances.

## Figures and Tables

**Figure 5:**
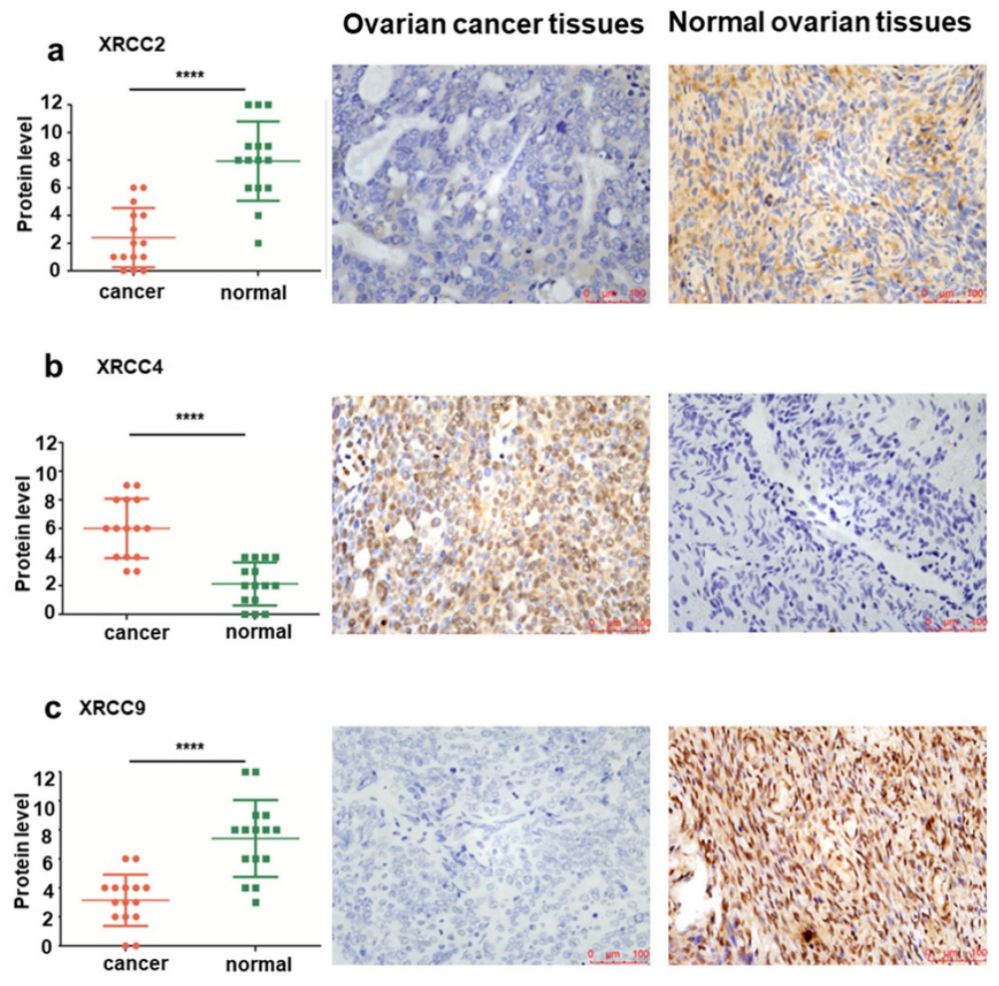
** The protein level and representative immunohistochemical staining images of XRCC2, XRCC4 and XRCC9 in tissues from ovarian carcinoma and normal ovary.** a-c: The protein levels of XRCC2, XRCC4 and XRCC9 were determined by immunohistochemical score in ovarian carcinoma samples and normal ovarian samples. The representative images of XRCC2, XRCC4 and XRCC9 were detected by IHC. **** *P* <0.0001. Data were triplicate and displayed as mean ± SD.

**Table 1:** Correlation of *XRCC2, XRCC4 and XRCC9* expression with PFS and OS in ovarian cancer patients with different pathological grades

XRCC	grades		PFS			OS	
		Cases	HR (95% CI)	*P* value	Cases	HR (95% CI)	*P* value
XRCC2	I	37	0.17 (0.05-0.56)	0.0009*	56	2.29(0.88-5.95)	0.08
	II	256	1.12 (0.81- 1.56)	0.48	324	1.34(0.97-1.85)	0.075
	III	837	0.76 (0.63- 0.91)	0.0026*	1015	0.8 (0.67-0.96)	0.016*
XRCC4	I	37	2.53 (0.82- 7.75)	0.093	56	3.92(1.42-10.85)	0.0049*
	II	256	0.76 (0.54- 1.06)	0.099	324	1.25 (0.88-1.76)	0.21
	III	837	1.38 (1.16- 1.65)	0.0004*	1015	1.56 (1.32-1.85)	0.0000*
XRCC9	I	37	2.47 (0.8 - 7.57)	0.1	56	2.02 (0.71-5.68)	0.18
	II	256	0.79(0.59 - 1.06)	0.12	324	0.9 (0.66-1.22)	0.51
	III	837	0.73(0.62 - 0.86)	0.0002*	1015	0.74 (0.62-0.87)	0.0003*

*P<0.05

**Table 2:** Correlation of *XRCC* gene expression level with PFS and OS in different clinical stage ovarian cancer patients

XRCC	Clinical stages	PFS			OS		
		Case	HR (95% CI)	*P* value	Case	HR (95%CI)	*P* value
XRCC2	I+II	163	2.11(1.19-3.76)	0.0093*	135	1.77(0.81-3.86)	0.15
	III+IV	1081	0.78 (0.67-0.9)	0.0010*	1220	0.72 (0.6-0.85)	0.0000*
XRCC4	I+II	163	1.77 (1-3.14)	0.046*	135	2.6 (1.2-5.65)	0.012*
	III+IV	1081	1.23(1.06-1.43)	0.0072*	1220	1.53(1.31-1.79)	0.0000*
XRCC9	I+II	163	1.6(0.88-2.89)	0.12	135	0.48 (0.21-1.11)	0.081
	III+IV	1081	0.77(0.67-0.88)	0.0003*	1220	0.79(0.68-0.92)	0.0022*

*P<0.05
